# Evaluation of China's public health system response to COVID-19

**DOI:** 10.7189/jogh.11.05004

**Published:** 2021-01-16

**Authors:** Pengfei Zhang, Jinghua Gao

**Affiliations:** School of Labor and Human Resources, Renmin University of China, Beijing, China

## Abstract

**Background:**

The focus of the study is to assess the advantages and shortcomings of China's public health system in the process of the COVID-19 prevention and to discuss the future reform of China's public health system.

**Methods:**

By searching literature and reports related to the COVID-19 prevention of China, we compared the prevention effectiveness with the prevention policies in the process of the COVID-19 prevention.

**Results:**

China's public health system can effectively combine national power to maximize the effectiveness of pandemic prevention. It improved the pandemic prevention ability of communities continuously and promoted the fairness of prevention. Traditional Chinese Medicine has also been used in pandemic prevention, which reduces the drug resistance of the virus. At the same time, the combination of the disease diagnosis and the Internet has reduced the spread speed of the pandemic. China's public health system also has some problems in response to the COVID-19, such as the shortage of medical resources, insufficient alerts, the low efficiency of reporting to superior government and the shortage of reward and punishment system for pandemic prevention.

**Conclusions:**

China's practice and efforts of the COVID-19 prevention can provide experience for other countries to improve their public health systems and accelerate the end of the COVID-19 pandemic.

In December 2019, several cases of pulmonary infection of unknown cause were found in Wuhan, Hubei Province, China, and gradually began to spread. The pathogen of the pulmonary infection was tentatively diagnosed as novel Coronavirus, On January 9, 2020 [1]. To prevent further spread of the pandemic, 31 Chinese provinces had launched the highest-level responses on January 29, 2020 [2]. WHO announced that the novel Coronavirus pandemic was listed as an international public health crisis on January 30, 2020. From February 8, 2020, the novel Coronavirus was defined as the COVID-19. Preventing further spread of COVID-19 is the key to winning the battle against the pandemic. China's public health system plays a vital role in the prevention of COVID-19. The outbreak of COVID-19 has seriously damaged the health of Chinese people and threatened their lives. Protecting the health of all citizens is the fundamental purpose of the construction of public health system in China.

After decades of exploration and improvement, China has gradually established a public health system which contains medical institutions and medical administrative institutions. Medical institutions include hospitals, clinics and community health centers. Medical administrative institutions are used to reform and improve public health laws and supervise the behavior of medical institutions. This article discusses the advantages and shortcomings of China's public health system in response to COVID-19. It also provides a reference for other countries to understand China's experience in pandemic prevention and has certain values for other countries' construction of public health system in response to COVID-19.

## METHODS

Through the qualitative design, based on the theoretical underpinning of the response of public health system to the pandemic, we compared the prevention effectiveness with the prevention policies in the process of the COVID-19 prevention by searching literature and reports related to the COVID-19 prevention of China.

A country's public health system plays a decisive role in pandemic prevention. Once a public health emergency occurs, the public health system needs to respond quickly to curb the widespread spread of the crisis [3]. However, when the characteristic of widespread crisis appears, it means that the public health system fails in the initial stage of pandemic prevention. At this time, while making up the failure loopholes, we should focus on the regional prevention of the pandemic, make full use of the medical resources among different regions, and minimize the losses of economic and social caused by the pandemic through efficient cooperation [4,5]. Therefore, how to realize the efficient response of the public health system to a pandemic is a hot issue studied by many scholars in the world.

According to the different development stages of the pandemic, there are some differences in the emphasis of the public health system [6]. It is embodied in the early stage of pandemic prevention and the response phase of the pandemic.

In the early stage of pandemic prevention, the public health system should have sufficient alerts [7]. Its main task is to find out the pandemic situation in a timely manner, understand relevant information through corresponding medical tests, and make an objective assessment of the spread speed and magnitude of the pandemic accordingly [8]. Sufficient alerts of pandemic are the starting point of the public health system, and the pandemic information captured is an important reference for pandemic prevention [9]. On the one hand, sufficient alerts can detect the pandemic in a timely manner, and on the other hand, sufficient alerts can obtain correct information about the outbreak [10]. The public health system should also have an efficient system of strategic decision and a preferential system of implementation based on the pandemic information [11]. Specifically, it involves how to timely disclose pandemic information, guide individual behavior, make corresponding deployment of pandemic prevention, and implement promptly and effectively actions [12]. The construction of the public health system focuses on the early stage can prevent the spread of the pandemic theoretically [13]. However, due to the suddenness and rapid spread of pandemic, it is difficult for us to keep track of the pandemic situation in time, which easily leads the incident to the response phase of pandemic [14]. This is also the reason why many countries focus their public health system on response phase of the pandemic.

In the response phase of pandemic, first of all, the public health system requires the government to maximize the mobilization of social forces and reduce the spread of the pandemic by promoting the extensive cooperation of members of the community, which is the basis for the overall control of the pandemic [15,16]. In order to control the spread of pandemic in the response phase, not only the government should take strong guidance measures, but also the members of the society should be required to cooperate closely [17]. This means that the state should formulate laws and regulations on pandemic prevention to clarify the rights of the government and the obligations of social members [18]. Second, because the response phase of pandemic is the stage of complete destruction of health, so it requires the public health system to fully mobilize medical resources and realize the coordinated allocation of medical resources among different regions [19-21].In particular, for areas with severe damage of pandemic, the supply of medical resources must be content to individual’s medical needs [22,23]. Due to the great differences in the transfer and receiving systems of medical resources among different regions, it is often difficult to carry out the plan of regional transfer of medical resources in reality. In areas where medical resources are scarce, it is extremely easy to cause huge casualties from pandemic [24]. Moreover, the order of social and economic should be restored in a timely manner after pandemic [25]. Therefore, the public health system should involve the system of society and economy reconstruction. The damage of society and economy caused by pandemic is obvious. Even if the pandemic is under control, it will not be a final victory. At this time, the focus should be on how to restore social stability and economic development as soon as possible, and ensure the normal work and normal life of individuals.

China's current public health system focuses more on the response phase of pandemic. It can be said that China's pandemic prevention has both advantages and shortcomings. Our article aims to evaluate China's public health system in responding to COVID-19, and to discuss the future reform of its health system. It is hoped that China's practice and efforts can provide experience for other countries to improve their public health systems, improve the effectiveness of response to the COVID-19.

## RESULTS

### Integration of public health system and national power

Integration of public health system and national power is a significant advantage in response to the COVID-19 in China. During the pandemic prevention, China's public health system had mobilized the initiative of governments and all sectors of society to take unified command, shadow the overall situation of pandemic, and racing against time to adapt to the development of the pandemic.

In the construction of medical institutions, through the integration of public health system and national power, China constructed many medical institutions in a short time. For example, with the reference of experience in previous pandemic prevention, China had built a “Huoshenshan Hospital” in 10 days. The area of “Huoshenshan Hospital” is more than 30 000 m^2^ and can receive about 5000 COVID-19 patients [26]. After the construction of “Huoshenshan Hospital”, China used the national power to build a lager medical institution named “Leishenshan Hospital”. The “Leishenshan Hospital” contains lots of medical equipment and can receive about 8000 COVID-19 patients [27].

Meanwhile, China used the national power to strengthened number of doctors to areas with severe situations of pandemic to avoid the shortage of medical personnel. China also used the national power to subsidize the equipment of pandemic prevention to society and help the social members to carry out the work of pandemic prevention. All the subsidization of pandemic prevention is from finance capital. As far as medical supplies are concerned, in order to ensure the sufficient supply of pandemic prevention, China used the national power to establish a temporary storage system of medical supplies. All the medical supplies contained in this system were produced by enterprises during the pandemic. In other words, China had used national power to help enterprises resume operation and produce medical supplies. In addition, China used the national power mobilize social members to donate medical supplies, and all the medical supplies from contribution were for pandemic prevention of communities.

### Improving the pandemic prevention ability of communities continuously

In the process of running the public health system, China attaches great importance to the development of medical institutes in communities. This is because the community is the most basic living unit, and medical needs of residents can be met efficiently though improving the medical ability of communities. China had been improving the pandemic prevention ability of communities continuously in the past few decades. For example, China has been increasing financial investment in medical institutions of communities, including introducing advanced medical equipment and high-level doctors into communities. Since 2005, the number of medical institutions in communities has risen from 849 500 to 943 600 in 2019. The number of community doctors has risen from 2.69/1000 in 2005 to 4.63/1000 in 2019. The number of community hospital bed has risen from 725 800 in 2005 to 1 583 600 in 2019. The allocation level of community medical resources has been greatly improved and the ability of pandemic prevention has been improved.

In the process of COVID-19 prevention, medical institutions of community have covered almost every corner of families. The pandemic prevention communities have many advantages. On the one hand, community propaganda about COVID-19 can improve the efficiency of pandemic prevention. It can strengthen community members' awareness of COVID-19, help to regulate the life behavior of community members and reduce the risk of pandemic infection. On the other hand, as an acquaintance society, the community is convenient for grid management and carpet investigation of pandemic prevention [28]. In this respect, the medical strength of community cannot only improve the management efficiency of pandemic prevention, but also ensure the accurate information of pandemic prevention. In addition, because of the advanced medical equipment and the high-level doctors, the community can receive some infection cases of COVID-19, which can reduce the risk of cross infection caused by the flow of infection cases.

The ability of community pandemic prevention is also reflected in the medical insurance system. China's public health system pays attention to the construction of community medical insurance system. Since 2010, China has formulated a health insurance system for community members, which is called “Basic Health Insurance System” (BHIS). The BHIS is a compulsory health insurance system, which requires every member of the community to join in. By 2019, the number of people participating in the BHIS had reached 897 million [29]. During the pandemic prevention, the BHIS can reduce the medical expenses of community members, and ensure the accessibility of their medical services.

### The aid of Traditional Chinese Medicine(TCM)

TCM is the crystallization of traditional wisdom formed in the course of fighting against diseases for more than 2000 years. With the development of TCM, the management efficiency of TCM has been continuously improved, and the technical level and service quality of TCM have changed significantly. China has put some TCM into the list of health insurance drugs since 2000, and the proportion of TCM in the list has been maintained about 50%. TCM occupies an important part in China's public health system. Since TCM was recognized by China's public health system, the number of TCM medicine institutions increased from 2453 in 2000 to 3977 in 2019. The number of visits in TCM medicine institutions reached 548 million in 2019, accounting for 15.32% of the total number of visits in medicine institutions. The number of hospital beds in TCM medicine institutions reached 0.84 million in 2019, accounting for 13.55% of the total number of hospital beds, of which the utilization rate of hospital beds in TCM medicine institutions reached 84.80%.

TCM plays an aid role in preventing pandemic and protecting the health of residents. In recent years, with the abuse of antibiotics, the drug resistance of bacteria and viruses has gradually increased, and the incidence of viral infections has increased year by year. In addition, the large-scale use of antibiotics has placed a serious burden on human body. Existing studies have shown that TCM which is based on pharmacological research and clinical syndrome has a large development space in anti-virus [30]. The biggest advantage of TCM in the treatment of COVID-19 patients is that it can enhance the immunity of patients and prevent the virus from producing drug resistance, which can make up for the shortcomings of Western Medicine. In the prevention of COVID-19, TMC was introduced into the treatment of patients at the first time in China. For example, on January 27, 2020, the “*Diagnosis and Treatment Plan of COVID-19*” promulgated by the China Administration of TCM announced the new TCM treatment plan for COVID-19 patients and extended it to the whole country. The therapeutic effect of TCM was optimistic, and the existing clinical cases and data showed that the symptoms of patients had been significantly improved after the treatment of TCM [31].

### The promotion of the Internet & Health Model (IMM)

The IMM takes the Internet as the carrier to provide real-time and convenient medical services to social members. With the development of information technology science, the Internet, as the carrier of public health system, has not only promoted the reform and improvement of China's medical instructions, but also accelerated the transformation from traditional treatment model to “online” treatment model. Compared with the traditional treatment model, the promotion of the IMM can decline the time cost of treatment and improve the efficiency of treatment because it can transmit the information of treatment through Internet. IMM can eliminate the unreasonable distribution of health resources among regions, enhance the utilization efficiency of health resources and improve the efficiency of medical services. Relevant studies have shown that the IMM can change traditional medical treatment methods, reconstruct medical treatment procedures, and lead the upgrading of the health industry. It not only has a subversive impact on the construction of China public health system, but also can force medical instructions reform [32]. This is because it can realize the vertical flow of high-quality health resources from developed areas to underdeveloped areas, and plays an important role in the treatment feeling of patients and alleviating the conflicting relation between doctors and patients [33].

IMM can improve the efficiency of China's public health system in preventing COVID-19. On the one hand, IMM can maximize the function of treatment cooperation between different regions through the “online” medical service. Especially for the areas with severe situations of pandemic, there is a lack of medical resources and technical personnel. Through IMM medical instructions can make the remote diagnosis and medical personnel can play a supporting role on treatments of other regions through the Internet information technology, which can reduce the burden of medical personnel in areas with severe situations of pandemic and make up for the shortage of health resources. On the other hand, for COVID-19 patients and suspected isolation cases, IMM can provide timely consultation and referral of medical information, which reduces the direct touch between medical personnel and patients, and effectively reduces the risk of cross infection. In addition, IMM can disclose pandemic information to social members in real time, which can promote the propaganda of pandemic situation and popularize the knowledge of pandemic prevention.

### The shortage of medical resources

In the short term, China's public health system is still unable to effectively deal with the shortage of medical resources, especially there are shortages of medical materials, medical facilities, hospital beds and technical personnel in areas with severe situations of pandemic. There are three reasons for the shortage of medical resources in areas with severe situations of pandemic. First, due to the serious pandemic situation, a large number of medical resources are needed in the process of COVID-19 prevention, and the actual demand is far more than the supply. Second, the severity of the COVID-19 increases the panic of residents, which easily leads residents to excessively hoard medical resources. As a result, the actual quantity of medical resources used is far more than the normal quantity, thus increasing the shortage of medical resources in areas with severe situations of pandemic. Third, the measures taken in the process of COVID-19 prevention in areas with severe situations of pandemic are more stringent. In order to reduce the mobility of personnel and prevent the spread of the pandemic, the entire region will suspend work and production, which will affect the production of medical resources, reducing the regional supplies of medical resources and causing the actual supplies of medical resources to be out of line with demands.

During the prevention of COVID-19, China's public health system lacks effective measures dealing with the supply-demand relationship of medical resources and coordinating the flow of medical resources among regions. Specifically speaking, on the one hand, China's public health system has not effectively allocated medical resources in areas with severe situations of pandemic, resulting in the shortage of hospital beds, long time in diagnosis of suspected case, excessive pressure of medical personnel. On the other hand, the public health system does not provide information of medical resource to residents timely, resulting in excessive purchase of medical resources, which not only enlarges the demand for medical resources, but also causes social unrest.

### Insufficient alerts

Sufficient alerts of pandemic plays an important role in pandemic prevention. Sufficient warning means that the key to pandemic prevention lies on efficient detection, judgment and information disclosure. Especially for the early stage of pandemic prevention, sufficient alerts can inhibit the development of the pandemic and reduce the spread speed of the pandemic. Because the pandemic has the characteristics of irregular outbreak, wide spread ways, high infectious and uncertain source of infection, insufficient alerts can increase the preparation time of pandemic prevention for medical instructions and social members, guide the production behaviors of enterprises and living behaviors of residents, which can reduce a part of the social loss. For the pandemic prevention, how to manage and use the ability of insufficient alerts is related to the effectiveness of prevention. Improving the ability of insufficient alerts can promote the detection and judgment of pandemic information by social members and reduce the hazards of pandemic from the origin.

China's public health system lacks the sufficient alerts in the process of COVID-19 prevention and the method of COVID-19 prevention is still limited to the traditional passive method. The important power of COVID-19 prevention comes from the social members, but the China's public health system did not provide them sufficient alerts, which forces them into a passive situation. In the early stage of COVID-19 prevention, because of the insufficient alerts, the detection method of medical institutions for social members was only the detection body temperature. They did not use efficient and accurate detection methods nucleic acid in time, which caused an increased risk of cross-infection faced by social members. When the COVID-19 completely broke out, because social members and medical instructions got little information about pandemic, the uncertainty of the pandemic information increased the panic of society and disturbs the social order, which virtually increases the social cost of COVID-19 prevention.

### The low efficiency of reporting to superior government

Timely reporting to superior government after discovering the pandemic can enable decision-makers to grasp the information of the pandemic situation at the first time. This is conducive to the decision-makers to monitor and scientifically determine the trend of the pandemic situation, and make timely decisions on pandemic prevention. Therefore, higher efficiency of reporting to superior government is very important for prevention in the response phase of pandemic.

The COVID-19 had revealed the low efficiency of reporting to superior government in the China's public health system. On the one hand, the process of pandemic reporting to superior government was not closely combined with informatics technology such as the Internet, which could not effectively use Internet to save the time cost of pandemic reporting, resulting in the low efficiency of reporting to superior government. For example, 27 cases of novel coronavirus pneumonia were found in early December 2019, which were later diagnosed as cases of the COVID-19, but the superior government did not receive relevant pandemic information until December 27, 2019. On December 31, 2019, the expert group of detecting pandemic arrived at the reporting area to carry out the work of verification, and on January 5, 2020, the work of verification was still in progress. All close contacts of COVID-19 cases began to be sequestered until January 11, 2020. It can be seen that the efficiency of reporting to superior government in China's public health system was too low, which led to the rapid spread of the pandemic, and the subsequent deployment work of the COVID-19 was disrupted. On the other hand, the regulations of reporting to superior government were too obsolete, which made it unable to adapt to the social development efficiently. For example, in 2003, China formulated “*Management Regulations for the Monitoring Pandemic* “, which were revised in 2006 and have been used today. The outstanding problems of these rules were that the process of reporting to superior government is too complex, and there was no effective interaction between the main bodies in the process of reporting. In a word, there is much improvement space in the management regulations for the monitoring pandemic of China's public health system.

### The shortage of punishment system for pandemic prevention

Reasonable punishment system can improve the efficiency of pandemic prevention, and stimulate the prevention autonomy of social members. Due to the sudden, widespread and destructive characteristics of the pandemic, it is urgent to prevent and control the pandemic situation. If there is negligent in the process of pandemic prevention, it is very likely to lead to disastrous consequences. If the efficiency of pandemic prevention is high, the pandemic situation can be controlled earlier, and huge losses will be reduced for national economic development and health of social members. Therefore, the perfect punishment system has important significance in pandemic prevention. Existing studies have shown that the shortage of punishment system in the process of pandemic prevention will lead to unclear responsibilities of prevention personnel [34]. The unclear responsibilities will lead various departments involved in pandemic prevention to shirk responsibilities, which reduces the coordination efficiency of pandemic prevention. Therefore, it is necessary to establish a punishment system in the public health system so as to clarify the tasks of various departments [35]. In this way, in the process of pandemic prevention, prevention personnel who have caused losses due to evasion of tasks can take corresponding responsibilities through the punishment system.

At present, the shortage of punishment system for pandemic prevention in China's public health system has led prevention personnel not to give full play to autonomy, and some work of the COVID-19 prevention cannot be put into practice because of unclear responsibilities. At the same time, the shortage of punishment system for pandemic prevention leads to the unclear responsibility boundary between the departments of the COVID-19 prevention and the overlapping of prevention tasks among administration departments. For example, in order to mobilize the enthusiasm of the COVID-19 prevention and improve the efficiency of prevention, some local governments were forced to formulate temporary punishment measures [32]. In addition, there were many intersecting responsibilities among administration departments in the process of the COVID-19 prevention, which has caused some local governments to be forced to make judgments on the departments' responsibilities.

## DISCUSSION

The COVID-19 has posed a huge challenge to China’s public health system. In order to respond more effectively to future outbreak of pandemic, China has to improve the public health system for pandemic prevention.

The local governments should pay special attention to the availability of treatment facilities, the number of hospital beds, and medical personnel in areas with severe situations of the COVID-19. Due to the strong time-effectiveness of medical supplies, the China’s public health system should be updated to improve the quality of medical supplies reserved in time based on ensuring abundance of medical supplies. In terms of the amount of medical resources, considering the level of local economic development and the number of permanent residents, local governments should reserve medical resources scientifically in a certain proportion according to the number of medical institutions and the level of medical consumption. It is necessary to avoid the shortage of medical resources caused by the low level of storage and the waste of medical resources caused by the excessive storage. In addition, the funds for medical resources reserved should be from country's finance.

China's public health system should ensure sufficient alerts in the process of the COVID-19. On the one hand, China's public health system should make social members, medical institutions and local governments enhance the awareness of the damage of the COVID-19. Especially for the areas where the situation of the COVID-19 is not serious, China's public health system should timely carry out reconnaissance and collect relevant information, and only in this way can we nip in the bud, which can get more time for the early prevention of the COVID-19. On the other hand, China's public health system should ensure the timely disclosure of the COVID-19 information and ensure that the whole development process of the COVID-19 is transparent to the public, so as to promote social members' participation in the pandemic prevention and control process. It is also important to improve the efficiency and accuracy of the COVID-19 information collection, because this can provide a lot of pandemic information for the social members in a short time, which can prepare social members for the COVID-19.

China's public health system should update the management regulations for the monitoring pandemic to improve efficiency of reporting to superior government. It is of significance to straighten out links of reporting to superior government and simplify the reporting process. To ensure that China's public health system can be respond to the COVID-19 in a timely and effective manner, medical instructions should abandon the way of paper documents in reporting to superior government, and make the Internet be used throughout process of reporting to superior government. For the regulations of monitoring pandemic, the China's public health system should allow any social member or medical personnel, not just medical institutions, to report the COVID-19 information directly to the decision-making administration departments, so as to ensure that the COVID-19 information obtained by the decision-making department is more accurate. Besides, it is necessary to improve the ability of information communication among all subjects in process of COVID-19 prevention. For medical personnel, when detecting an unknown virus, they should actively communicate and coordinate with the superior medical administrative department. For the local government, it is necessary to deal with the reported information of COVID-19 in time and send the processed results to the social members.

Moreover, the COVID-19 prevention needs the joint efforts of multiple actors and trans-department coordination and any problem in any link may lead to the failure of the COVID-19 prevention. Therefore, China's public health system should establish the punishment system, which can clarify the specific responsibilities of each subject in the process of COVID-19 prevention. This is not only conducive to improving the autonomy of prevention personnel, but also improving the efficiency of COVID-19 prevention.

## CONCLUSION

Through the evaluation of China's public health system in response to the COVID-19, this article found the advantages and shortcomings of China's public health system in the process of COVID-19 prevention, which has strong theoretical significance and practical value for other countries. Other countries' public health systems should also consider absorbing national power when responding to the COVID-19, and maximize the effectiveness of the COVID-19 prevention. At the same time, they should consider improving the COVID-19 prevention ability of communities continuously, so as to promote the fairness of prevention. They can consider the aid of Traditional Chinese Medicine, which can reduce the drug resistance of the COVID-19. In addition, they should combine disease diagnosis with the Internet in the process of pandemic prevention, which helps to avoid disorderly gathering of patients and slows down the spread of the COVID-19. Of course, China's public health system should also deal with the problems of the shortage of medical resources, insufficient alerts, the low efficiency of reporting to superior government, and the shortage of punishment system for pandemic prevention. These problems should also be considered in other countries during the COVID-19 prevention.
